# Fracture reduction by postoperative mobilisation for the treatment of hyperextension injuries of the thoracolumbar spine in patients with ankylosing spinal disorders

**DOI:** 10.1007/s00402-017-2653-7

**Published:** 2017-02-21

**Authors:** Richard A. Lindtner, Christian Kammerlander, Michael Goetzen, Alexander Keiler, Davud Malekzadeh, Dietmar Krappinger, Rene Schmid

**Affiliations:** Department of Trauma Surgery, Medical University of Innsbruck, Anichstraße 35, 6020 Innsbruck, Austria

**Keywords:** Spinal fractures, Ankylosing spondylitis, Diffuse idiopathic skeletal hyperostosis, Ankylosing spinal disorders, Hyperextension injury, Extension distraction injuries, Fracture reduction, Thoracolumbar spine, Posterior instrumentation, Percutaneous fixation, Outcomes

## Abstract

**Introduction:**

The aim of this study was to evaluate results of surgical stabilisation of hyperextension injuries of the thoracolumbar spine in patients with ankylosing spinal disorders using two different treatment strategies: the conventional open rigid posterior instrumentation and percutaneous less rigid posterior instrumentation. Surgical and non-surgical complications, the postoperative radiological course, and clinical outcome at final follow-up were comparatively assessed. Moreover, we sought to discuss important biomechanical and surgical aspects specific to posterior instrumentation of the ankylosed thoracolumbar spine as well as to elaborate on the advantages and limitations of the proposed new treatment strategy involving percutaneous less rigid stabilisation and fracture reduction by postoperative mobilisation.

**Materials and methods:**

Between January 2006 and June 2012, a consecutive series of 20 patients were included in the study. Posterior instrumentation was performed either using an open approach with rigid 6.0 mm bars (open rigid (OR) group) or via a percutaneous approach using softer 5.5 mm bars (percutaneous less rigid (PLR) group). Complications as well as the radiological course were retrospectively assessed, and patient outcome was evaluated at final follow-up using validated outcome scores (VAS Spine Score, ODI, RMDQ, Parker Mobility Score, Barthel Score and WHOQOL-BREF).

**Results:**

Surgical complications occurred more frequently in the OR group requiring revision surgery in two patients, while there was no revision surgery in the PLR group. The rate of postoperative complications was lower in the PLR group as well (0.7 vs. 1.3 complications per patient, respectively). Fracture reduction and restoration of pre-injury sagittal alignment by postoperative mobilisation occurred within the first 3 weeks in the PLR group, and within 6 months in the OR group. The clinical outcome at final follow-up was very good in both groups with no relevant loss in VAS Spine Score (pain and function), Parker Mobility Score (mobility), and Barthel Index (social independency) compared to pre-operative values.

**Conclusions:**

This study indicates that the proposed treatment concept involving percutaneous less rigid posterior instrumentation and fracture reduction by postoperative mobilisation is feasible, seems to facilitate adequate reduction and restoration of pre-injury sagittal alignment, and might have the potential to reduce the rate of complications in the management of hyperextension injuries of the ankylosed thoracolumbar spine.

## Introduction

Ankylosing spinal disorders, i.e., ankylosing spondylitis (AS) and diffuse idiopathic skeletal hyperostosis (DISH), are diseases of unknown aetiology, which lead to ankylosis of the spine in later stages [1]. This massively alters the biomechanics of the spine by eliminating the segmental elasticity provided by discs and ligaments [2]. Patients with ankylosing spinal disorders are prone to sustain spinal injuries even after low-energy trauma due to long-lever arms for any forces to act on the rigid yet brittle spine [3–5]. The ankylosed spine is unable to dissipate the energy impact to adjacent motion segments and therefore behaves biomechanically more akin to the diaphyseal part of a long bone.

Different biomechanical properties of the ankylosed spine inevitably lead to different fracture patterns as well. While hyperextension injuries of the thoracolumbar spine are uncommon in the entire population, they represent the predominant fracture pattern in patients with ankylosing spinal disorders [3, 5–7]. These fractures typically involve both the anterior and posterior columns of the spine and are, therefore, regarded as unstable injuries requiring surgical stabilisation [5, 6, 8]. Combined posterior–anterior stabilisation is widely considered to be the optimal treatment for cervical spine fractures in these patients [2, 4, 9]. The treatment of thoracolumbar fractures is described in a few studies and generally involves posterior instrumentation only [3, 4, 6, 9–11]. These studies, however, solely focus on the description of patients' characteristics without including a control group or discussing biomechanical and surgical aspects of posterior instrumentation in the ankylosed thoracolumbar spine.

The aim of this study, therefore, was to evaluate clinical and radiological outcome after surgical stabilisation of hyperextension injuries of the thoracolumbar spine in patients with ankylosing spinal disorders using two different treatment strategies: the conventional open rigid posterior instrumentation as well as a new treatment concept involving percutaneous less rigid posterior instrumentation and fracture reduction by postoperative mobilisation. The rationale behind the latter treatment strategy was to (1) enable adequate fracture reduction via postoperative mobilisation as adequate intraoperative reduction is exceedingly difficult and often impossible to accomplish in a prone position in these injuries and to (2) reduce the rate of wound healing complications and infections frequently encountered in these patients. Moreover, we sought to discuss important aspects specific to posterior instrumentation of the ankylosed thoracolumbar spine as well as to elaborate on the advantages and limitations of the proposed new treatment concept.

## Methods

The study was approved by the institutional review board and written informed consent was obtained from all patients. Inclusion criteria were defined as follows: (1) unstable hyperextension fracture of the thoracolumbar spine (type B3 according to Magerl) after low-energy trauma involving the anterior and posterior column, (2) ankylosing spinal disorder (i.e., AS or DISH, with all patients either previously diagnosed, or meeting diagnostic criteria for AS [12] or DISH [13]) with at least three ankylosed segments both cranially and caudally of the fracture, and (3) ability to walk without walking aids prior to the injury. Exclusion criteria comprised (1) hyperextension injuries of the thoracolumbar spine in patients without ankylosing spinal disorders or after high-energy trauma, (2) stable fractures involving one column of the spine only, (3) neurological deficits at admission or at discharge, and (4) concomitant fractures.

At admission, X-rays in a supine position and computed tomography (CT) scans were performed and neurological impairment was excluded in all patients. The fracture level and the fracture pattern were determined on the pre-operative CT scan. The fracture levels were classified as fractures of the thoracic spine (Th1–Th10), the thoracolumbar junction (Th11–L2), and the lumbar spine (L3–L5). Four fracture patterns were distinguished according to the fracture course through the anterior column (type 1: disc; type 2: vertebral body; type 3: anterior body and posterior disc; type 4: anterior disc and posterior body). The fracture displacement was assessed by measuring lordotic angulation, translation, and distraction of the fracture. Lordotic angulation was described with positive values. Translation was defined as the sagittal displacement of the posterior wall cranially and caudally of the fracture. Distraction was defined as the closest distance of the fracture gap perpendicular to the endplates. Pre-operative co-morbidities were assessed using the Charlson Comorbidity Index.

All surgeries were performed by two of the authors (RS, DK). Posterior instrumentation without fusion was performed with the patients in a flat prone position using two different pedicle screw-based systems with screws inserted bilaterally in either two or three segments both cranially and caudally of the fracture; the number of pedicle screws used did not differ between the two treatment groups (Table 2). There was no randomisation. The type of posterior instrumentation and optional cement augmentation of the pedicle screws was chosen by the individual surgeon. For the conventional open rigid posterior instrumentation (OR group), USS™ Low Profile Pedicle Screw System (Synthes, Oberdorf, Switzerland) was inserted via a standard open posterior midline approach. Side-loading monoaxial screws with a diameter of 6 mm and rods with a diameter of 6 mm composed of a Titanium alloy (TAN, ultimate tensile strength of 1060 MP) [14] were used. The ultimate tensile strength (UTS) is the maximum stress that a material can withstand per mm^2^ of cross-section area while being stretched before failure (i.e. plastic deformation in ductile and breakage in brittle materials) occurs. This results in a maximum load of 29.97 kN (1060 MP × 28.27 mm^2^ cross-section area), until plastic deformation of the USS rods occurs. For percutaneous less rigid posterior instrumentation (PLR group), CD Horizon Longitude™ Multilevel Percutaneous Fixation System (Medtronic, Memphis, TN, USA) was inserted percutaneously using bilateral stab incisions without actively performing intraoperative fracture reduction. Top-loading screws with variable axis screw heads and a diameter of 5.5 mm were used. The less rigid rods have a diameter of 5.5 mm and were composed of commercially pure Titanium (TiCP, UTS 860 MP). The maximum load of these rods is 20.43 kN (860 MP × 23.76 mm^2^), until plastic deformation occurs.

The radiological follow-up included X-rays in a supine position as well as CT scans immediately postoperative prior to mobilisation. CT scans were used to assess pedicle screw misplacement according to Abul-Kasim et al. [15] and cement extravasation. Additional X-rays were performed after mobilisation, at 3 weeks and at 3, 6, and 12 months after surgery in a standing position.

Surgical and non-surgical complications as well as length of hospital stay were retrospectively assessed. At final follow-up, the clinical outcome was assessed using the following validated questionnaires related to spinal injury: Visual Analogue Scale (VAS) Spine Score, Roland and Morris Disability Questionnaire (RMDQ), Oswestry Disability Index (ODI), and the abbreviated WHO Quality of Life questionnaire (WHOQOL-BREF). The geriatric assessment included the Barthel Index and the Parker Mobility Score. The patients were additionally asked to complete three scores (VAS Spine Score, Barthel Index, and Parker Mobility Score) to the best of their knowledge for the time prior to the injury to assess impairment in back-specific pain and function (VAS Spine Score), social dependency (Barthel Index), and mobility (Parker Mobility Score) associated with the injury.

SPSS 16.0 (SPSS Inc., Chicago, IL) was used for statistical analysis. Metric scaled data are reported as arithmetic mean ± standard deviation and categorical data as absolute frequency and percentage distribution. Depending on the distribution form, a *t* test for independent variables or a nonparametric Mann–Whitney *U* test was used to compare the two treatment groups. The distribution form was determined using the Kolmogorov–Smirnov test. A Chi-Square test or a Fisher Exact test was used for analysis of categorical data. The probability level was set at *p* < 0.05.

## Results

Between January 2006 and June 2012, a consecutive series of 20 patients met the inclusion criteria and were included in the study after obtaining written informed consent. There were 14 patients in the OR group and six patients in the PLR group. The demographical and injury-related data (Table 1) did not significantly differ between the two study groups (*p* > 0.05). None of the patients in the PLR group required conversion to an open approach. Surgery-related data are shown in Table 2. The relative frequency of pedicle screw misplacement did not significantly differ between the two groups (*p* > 0.05). There were no clinically relevant complications due to screw misplacement or cement extravasation. We observed five surgical complications in the OR group (pedicle screw loosening in two cases and impaired wound healing in three cases), which required revision surgery in two patients. Postoperative non-surgical complications are shown in Table 3. The number of complications was higher in the OR group (*p* > 0.05). More than half of all patients (11/20) had postoperative pulmonary complications. One patient from the OR group died on day 13 after surgery due to sepsis and multiple organ dysfunction syndrome (MODS). The length of hospital stay was 22.3 (±21.0) days in the OR group and 16.3 (±6.5) days in the PLR group (*p* > 0.05). Table 4 displays the radiological follow-up data for lordotic displacement. The posttraumatic, intraoperative, and postoperative lordotic angles prior to mobilisation were comparable between the two groups (*p* > 0.05). In the PLR group, the mean lordotic angle decreased from 6.5° (±4.9°) in the postoperative X-ray to 0.7° (±0.8°) after 3 weeks. In the OR group, the mean lordotic angle continuously decreases within the first 6 postoperative months, resulting in significant differences between the two groups at 3 weeks and at 6 months (*p* < 0.05). The mean posttraumatic translational displacement was 0.6 (±1.2) mm in the OR group and 0.7 (±1.0) mm in the PLR group, while distraction was 0.8 (±1.4) mm and 1.1 (±1.5) mm, respectively. The postoperative translational displacement was 0.6 (±1.0) and 0.4 (±1.0), and the mean postoperative distraction was 0.6 (±1.6) mm and 0.5 (±1.2.) mm, respectively. The values for translational displacement and distraction were, therefore, very low and remained constant in the further course. No neurological complications were encountered in any of the patients. The final clinical follow-up examination was performed after a mean of 29.2 (12–98) months (Table 5). There were no significant differences in any of the clinical outcome measures between the two groups (*p* > 0.05). The loss in VAS Spine Score, Barthel Index, and Parker Mobility Score between the pre-traumatic level and the level at final follow-up was only minimal in both groups (Table 5).

**Table 1:** Demographical and injury-related data

	All (*n* = 20)	OR group (*n* = 14)	PLR group (*n* = 6)	*p* value
Age	74.7 ± 10.9	76.4 ± 11.4	70.6 ± 9.2	0.28
Sex				
Male	18	12	6	0.99
Female	2	2	0	
Charlson comorbidity index	1.8 ± 1.9	1.4 ± 1.5	2.7 ± 2.5	0.18
Injury region				
Thoracic spine	6	3	3	0.30
Thoracolumbar junction	14	11	3	
Lumbar spine	0	0	0	
Fracture pattern				
Disc	6	4	2	0.98
Vertebral body	6	4	2	
Anterior body, posterior disc	4	3	1	
Anterior disc, posterior body	4	3	1	

**Table 2:** Surgery-related data

	All (*n* = 20)	OR group (*n* = 14)	PLR group (*n* = 6)	*p* value
Number of pedicle screws	8.8 (8–12)	8.9 (8–12)	8.3 (8–10)	0.28
% Screw misplacement	10.3 ± 14.4	9.2 ± 13.4	12.5 ± 17.7	0.78
Cement augmentation				
Yes	9	8	1	
No	11	6	5	
Cement extravasation				
Yes	6	5	1	
No	3	3	0	
Loosening of pedicle screws				
Yes	2	2	0	
No	18	12	6	
Impaired wound healing				
Yes	3	3	0	
No	17	11	6	

**Table 3:** Non-surgical postoperative complications

	All (*n* = 20)	OR group (*n* = 14)	PLR group (*n* = 6)	*p* value
Number of complications per patient	1.1 (0–3)	1.3 (0–3)	0.7 (0–2)	0.25
Pulmonary complications	11	10	1	
Urinary tract infection	6	4	2	
Delirium	1	1	0	
Decubital ulcera	2	1	1	
Sepsis/MODS	2	2	0	

**Table 4:** Radiological follow-up of lordotic angulation

	All (*n* = 20)	OR group (*n* = 14)	PLR group (*n* = 6)	*p* value
Lordotic angulation (°)				
Trauma	8.1 ± 5.5	8.0 ± 4.8	8.3 ± 7.3	0.92
Intraoperative	5.8 ± 4.4	5.2 ± 4.2	7.0 ± 5.1	0.47
Postoperative	5.1 ± 4.5	4.5 ± 4.4	6.5 ± 4.9	0.41
3 Weeks	2.9 ± 3.3	3.8 ± 3.6	0.7 ± 0.8	0.01*
3 Months	1.8 ± 1.9	2.4 ± 2.0	0.3 ± 0.5	0.02*
6 Months	0.6 ± 1.2	1.0 ± 1.7	0.2 ± 0.4	0.35
1 Year	0.5 ± 1.2	1.0 ± 1.7	0.0 ± 0.0	0.27

*p < 0.05

**Table 5:** Clinical results at final follow-up

	All (*n* = 20)	OR group (*n* = 14)	PLR group (*n* = 6)	*p* value
VAS Spine Score before trauma (0–100; 100 = no complaints/pain)	91.7 ± 15.6	85.0 ± 20.5	98.4 ± 2.8	0.17
VAS Spine Score at final follow-up (0–≥100; 100 = no complaints/pain)	89.6 ± 16.7	84.1 ± 21.4	95.2 ± 9.2	0.28
Loss in VAS Spine Score	2.1 ± 4.8	0.9 ± 1.2	3.2 ± 6.7	0.45
Roland and Morris Disability Questionnaire (0–24; 0 = no complaints/pain)	1.5 ± 2.2	1.3 ± 2.4	1.7 ± 2.1	0.80
Oswestry Disability Index (0–100; 0 = no complaints/pain)	5.7 ± 8.9	6.8 ± 11.5	4.7 ± 6.3	0.70
WHOQOL-BREF Overall Quality of Life (4–20; 20 = best value)	14.3 ± 2.7	14.7 ± 2.1	14.0 ± 3.3	0.69
WHOQOL-BREF General Health (4–20; 20 = best value)	15.0 ± 3.9	16.0 ± 4.4	14.0 ± 3.3	0.40
WHOQOL-BREF Physical Health (4–20; 20 = best value)	16.5 ± 2.1	17.5 ± 1.2	15.5 ± 2.4	0.11
WHOQOL-BREF Psychological Health (4–20; 20 = best value)	16.8 ± 1.8	17.3 ± 2.3	16.2 ± 1.0	0.27
WHOQOL-BREF Social Relationships (4–20; 20 = best value)	17.5 ± 2.3	17.6 ± 2.3	17.3 ± 2.6	0.86
WHOQOL-BREF Environment (4–20; 20 = best value)	18.9 ± 1.7	18.8 ± 1.9	18.8 ± 1.6	0.99
Parker Mobility Score before trauma (0–9; 9 = independently mobile)	8.7 ± 0.8	9.0 ± 0.0	8.3 ± 1.0	0.18
Parker Mobility Score at final follow-up (0–9; 9 = independently mobile)	8.3 ± 1.0	8.7 ± 0.8	7.8 ± 1.0	0.14
Loss in Parker Mobility Score	0.4 ± 0.8	0.3 ± 0.8	0.5 ± 0.8	0.73
Barthel Index before trauma (0–100; 100 = socially independent)	98.8 ± 4.3	100.0 ± 0.0	97.5 ± 6.1	0.36
Barthel Index at final follow-up (0–100; 100 = socially independent)	98.0 ± 4.4	99.2 ± 2.0	96.8 ± 5.9	0.38
Loss in Barthel Index	0.8 ± 1.5	0.8 ± 2.0	0.7 ± 1.0	0.86

## Discussion

In this study, we assessed clinical and radiological outcomes after surgical stabilisation of hyperextension injuries of the ankylosed thoracolumbar spine by either the conventional open rigid posterior instrumentation or by following a new treatment concept involving percutaneous less rigid posterior instrumentation and fracture reduction by postoperative mobilisation. The latter treatment strategy was developed in an attempt to (1) improve efficiency of fracture reduction and restoration of pre-injury sagittal alignment to ensure osseous contact at the fracture site which is a prerequisite for osseous healing, as well as to (2) reduce the rate of wound healing complications and infections frequently encountered in these patients, probably as a consequence of atrophic and degenerative trunk muscles resulting from muscle inactivity due to spinal ankylosis.

Spinal fractures typically involve the motion segment (i.e. discs, facet joints and ligaments) and are therefore regarded as articular fractures. Since a reconstruction of the motion segment is not feasible given the available treatment options, the surgical treatment of these injuries generally requires fusion of the motion segment. In the ankylosed spine, however, the motion segments have already spontaneously fused and the spine acts biomechanically more like the diaphyseal part of a long bone. The aim of the surgical treatment of fractures of the ankylosed spine are therefore similar to those of shaft fractures and include reduction and stabilisation of the fracture in order to promote osseous healing [2, 6].

At first glance, plate fixation via an anterior approach seems to be the best option for both reduction and stabilisation of hyperextension injuries of the ankylosed thoracolumbar spine. The exposure provided by the anterior approach may allow for direct reduction of the hyperextension fracture, while anterior plate fixation biomechanically acts as a tension band for the neutralisation of extension forces. Anterior approaches and plate fixation, however, have some major drawbacks in these patients. First, osteoporosis is frequently associated with ankylosing spinal disorders [1, 4, 8, 16] and reduces the strength of screw anchorage. Second, long-lever arms lead to high torques in the ankylosed spine. These moments have to be neutralized by the implant, until healing has occurred. Both facts highly increase the risk of failure of the bone–implant interface with subsequent implant loosening. The use of longer plates with more points of fixation reduces the risk of implant loosening. It may, however, be hard to implant longer plates via an anterior approach in the presence of a rigid thorax and pre-existing kyphosis [2]. In addition, peri- and postoperative pulmonary complications are frequently observed in these patients [7, 9, 17]. In our study, more than half of all patients developed postoperative pulmonary complications. This additionally argues against an anterior approach.

**Fig. 1:**
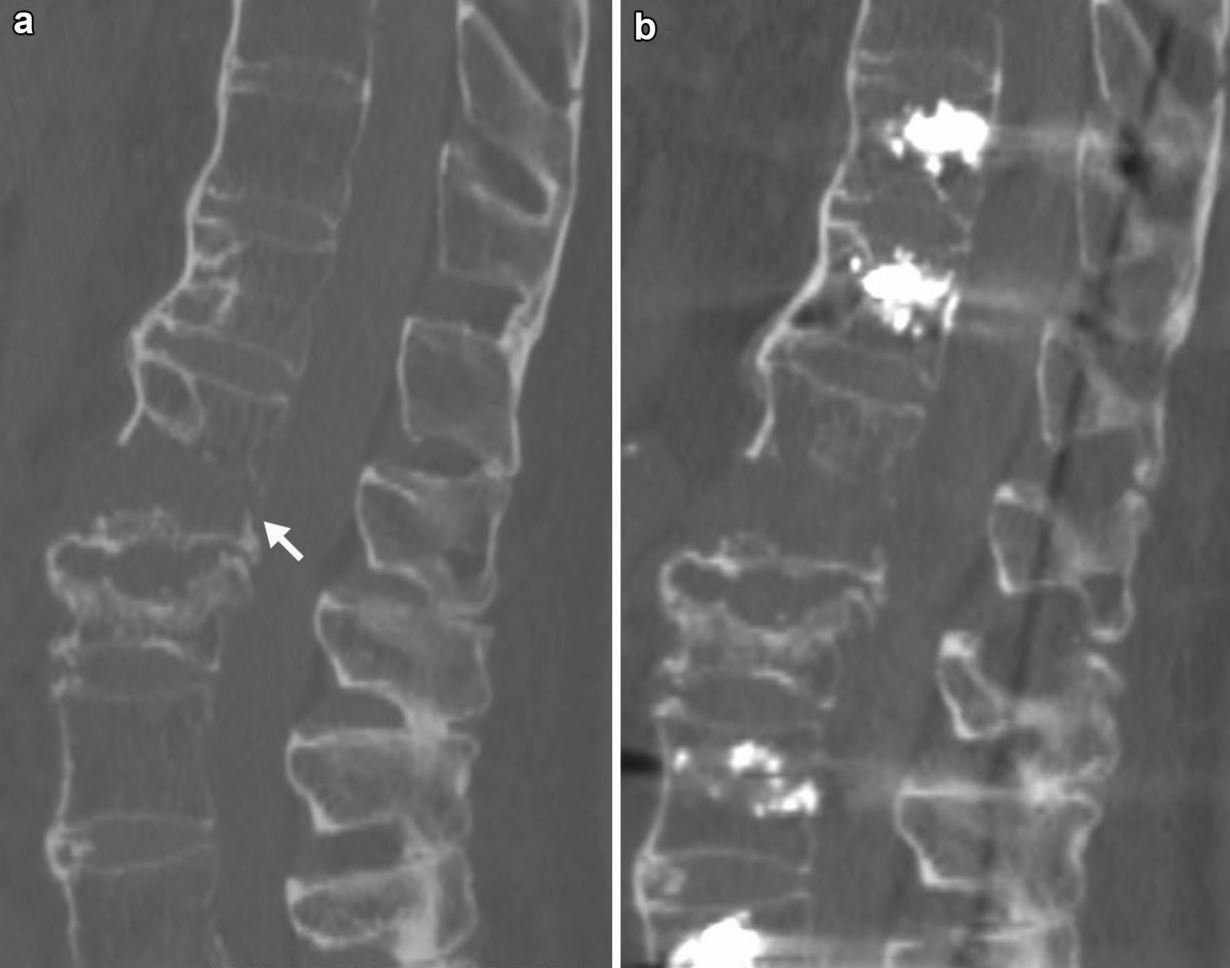
79-year-old male, hyperextension injury of Th12 (above a pre-existing, healed compression fracture of L1), 21° of lordotic angulation in the pre-operative CT scan (**a**). Postoperative CT scan prior to mobilisation after cement-augmented percutaneous less rigid posterior instrumentation. No relevant intraoperative reduction of the lordotic angulation (**b**)

**Fig. 2:**
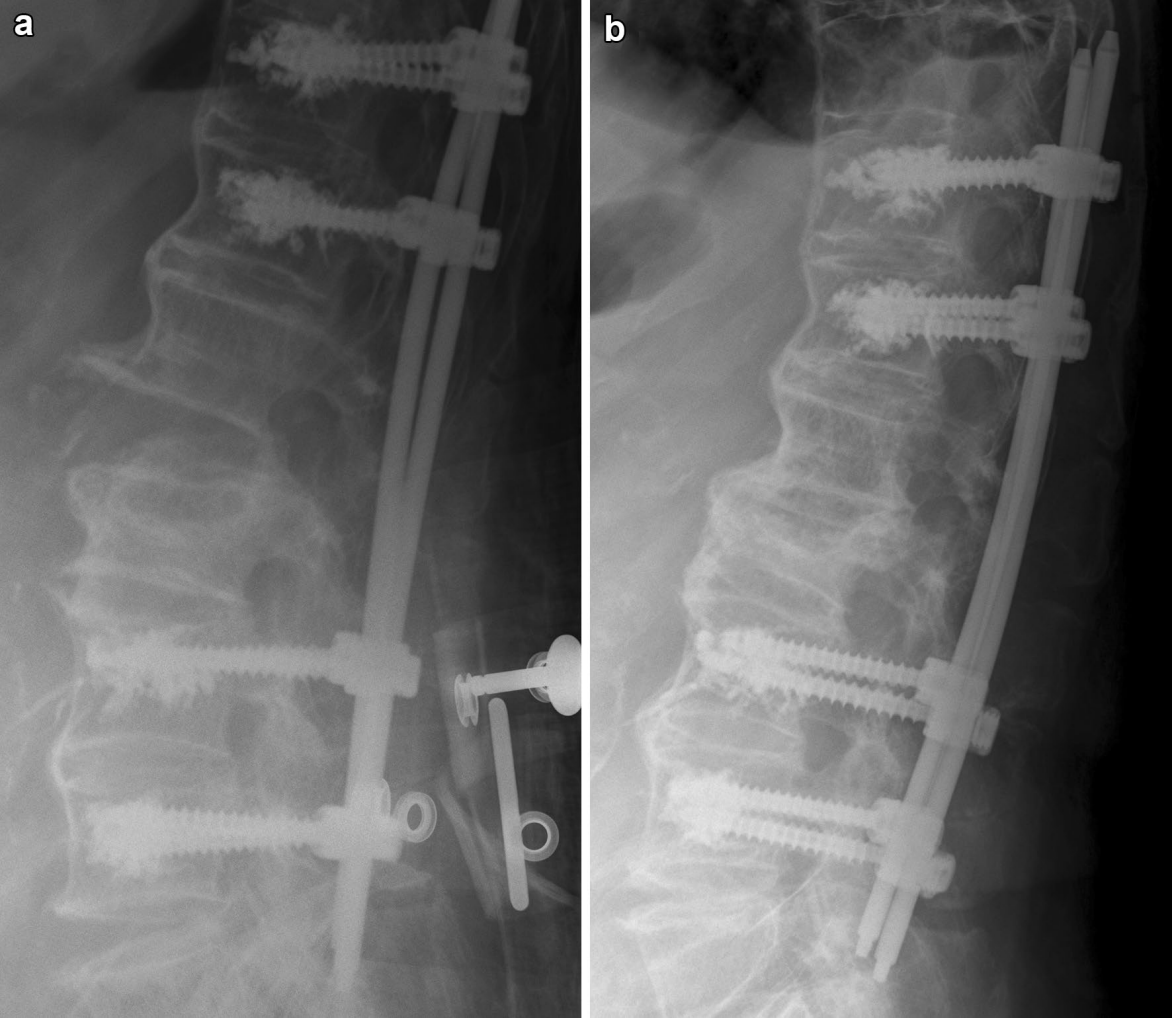
Postoperative X-ray control of the same patient prior to mobilisation (**a**) and after three weeks (**b**). Fracture reduction by postoperative mobilisation due to bending of the rods and restoration of pre-injury sagittal alignment

Posterior instrumentation is, therefore, regarded as the standard treatment for hyperextension injuries of the thoracolumbar spine in patients with ankylosing spinal disorders [3, 4, 6, 9]. Implant loosening, however, is a major issue in these patients after posterior instrumentation as well with rates reported as high as 15% [18]. There are several options to reduce the risk of implant loosening. First, cement augmentation of the pedicle screws increases the strength of the bone-implant interface. Second, multisegmental posterior instrumentation distributes the entire load to more points of fixation [19]. The third option is the reduction of the rigidity of the instrumentation. Less rigid constructs absorb part of the energy during mobilisation and, therefore, decrease the strain at the bone-metal-interface. The rigidity of the posterior instrumentation may be reduced by both increasing the working distance of the rods (i.e. the distance between the pedicle screws adjacent to the fracture) [19] as well as by reducing the bending stiffness of the longitudinal rods [20]. In our study, we used two rods of different bending stiffness. We observed no implant loosening in the group with softer rods (PLR group) despite a lower rate of cement augmentation of the pedicle screws (1/6 cases) compared to two cases of pedicle screw loosening in the OR group (cement augmentation in 8/14 cases, Table 2). We, therefore, advocate to reduce the rigidity of the posterior instrumentation by using soft rods. In addition, the working distance of the rods may be increased by leaving at least one vertebral body adjacent to the fracture site without pedicle screws, even if the fracture type would allow the insertion of pedicle screws in all vertebral bodies (Figs. 1b, 2). Moreover, considering the high rate of postoperative wound complications reported for patients with ankylosing spinal disorders (see below), using a percutaneous less rigid instrumentation, and optionally pedicle screw cement augmentation seems to be more favourable to reduce the risk of implant loosening than excessively increasing the instrumentation length at the risk of soft tissue complications.

**Fig. 3:**
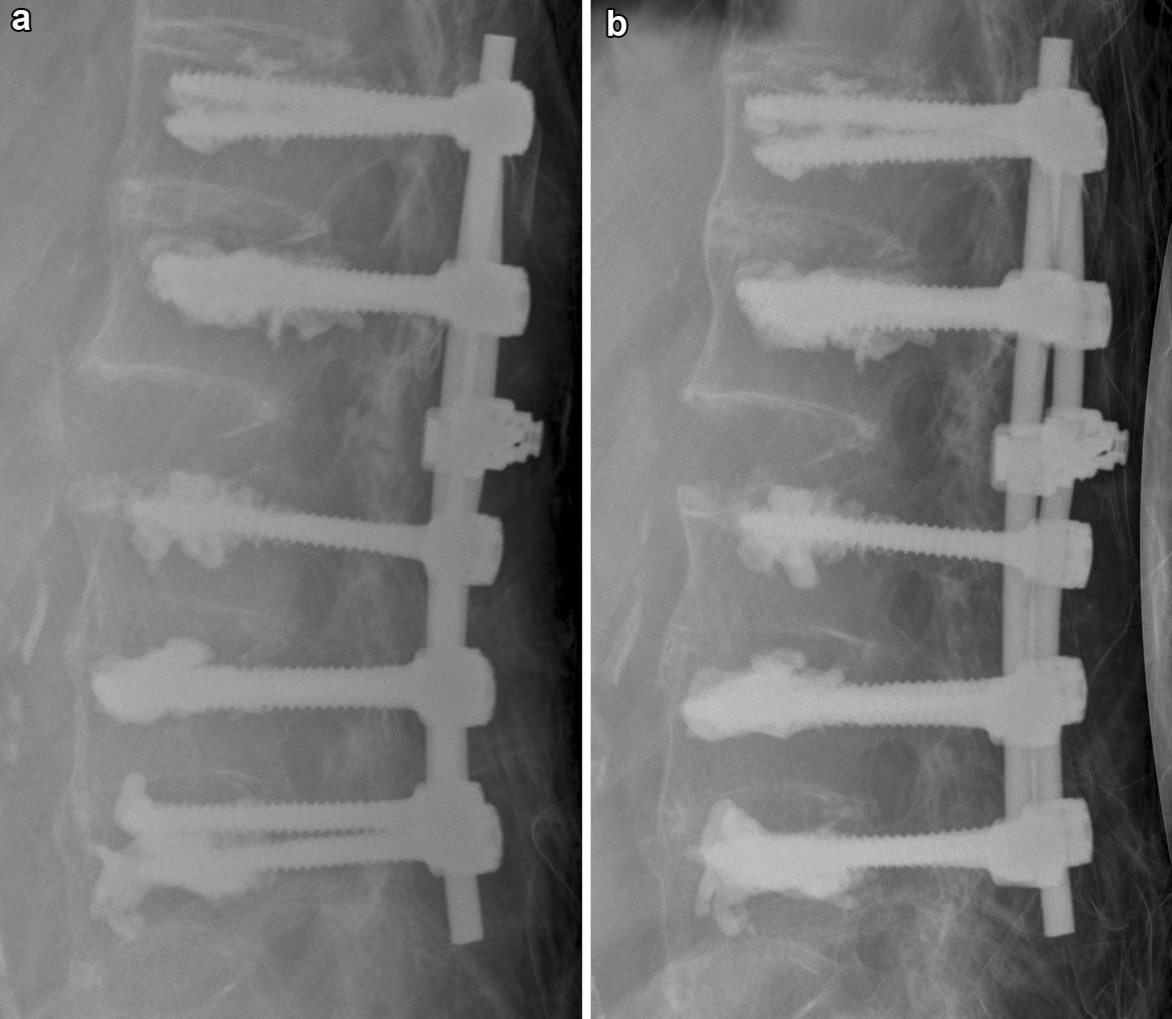
Postoperative X-ray control of the same patient prior to mobilisation (**a**) and after 3 weeks (**b**). No restoration of pre-injury sagittal alignment

**Fig. 4:**
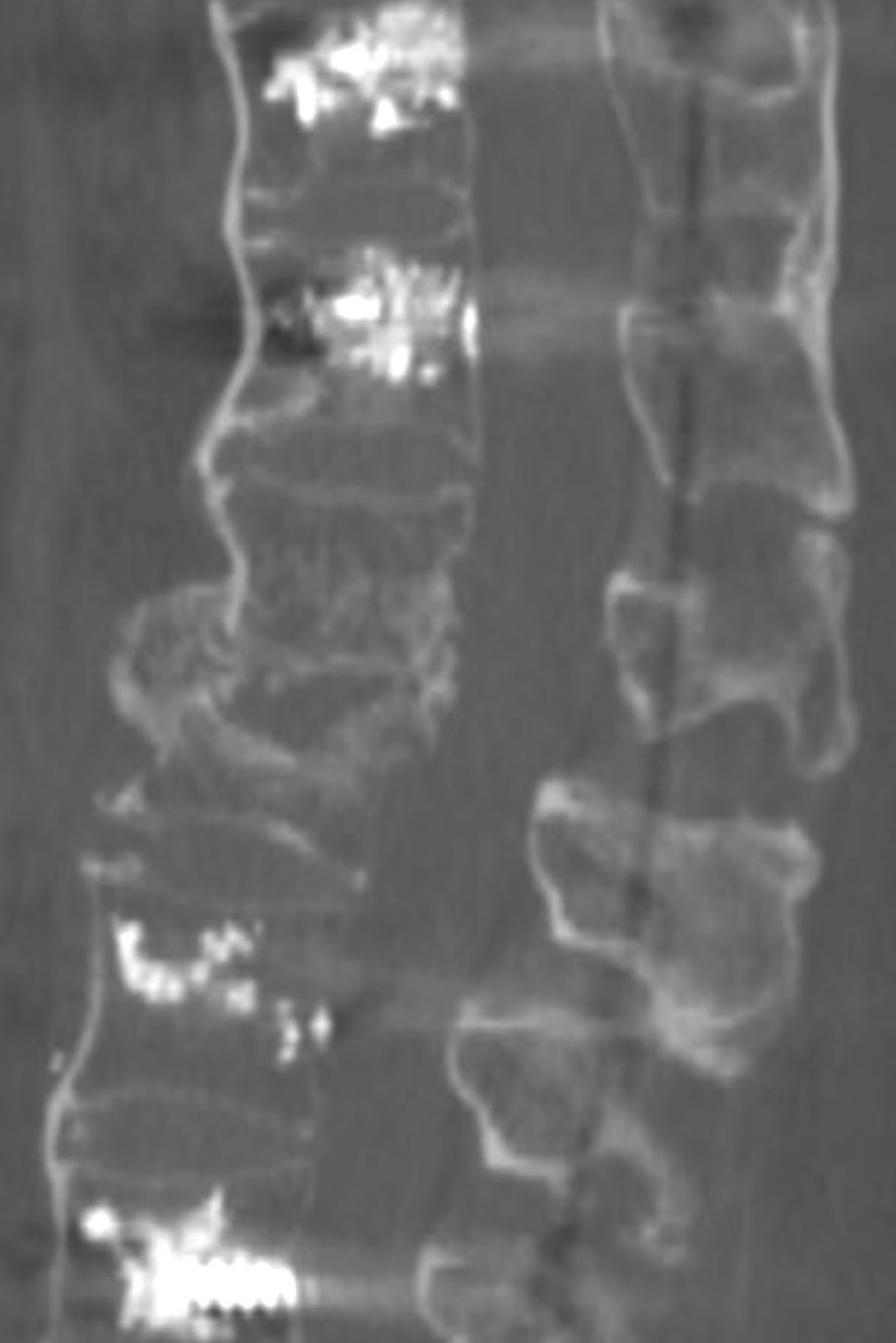
CT scan after 6 months showing osseous healing of the fracture

**Fig. 5:**
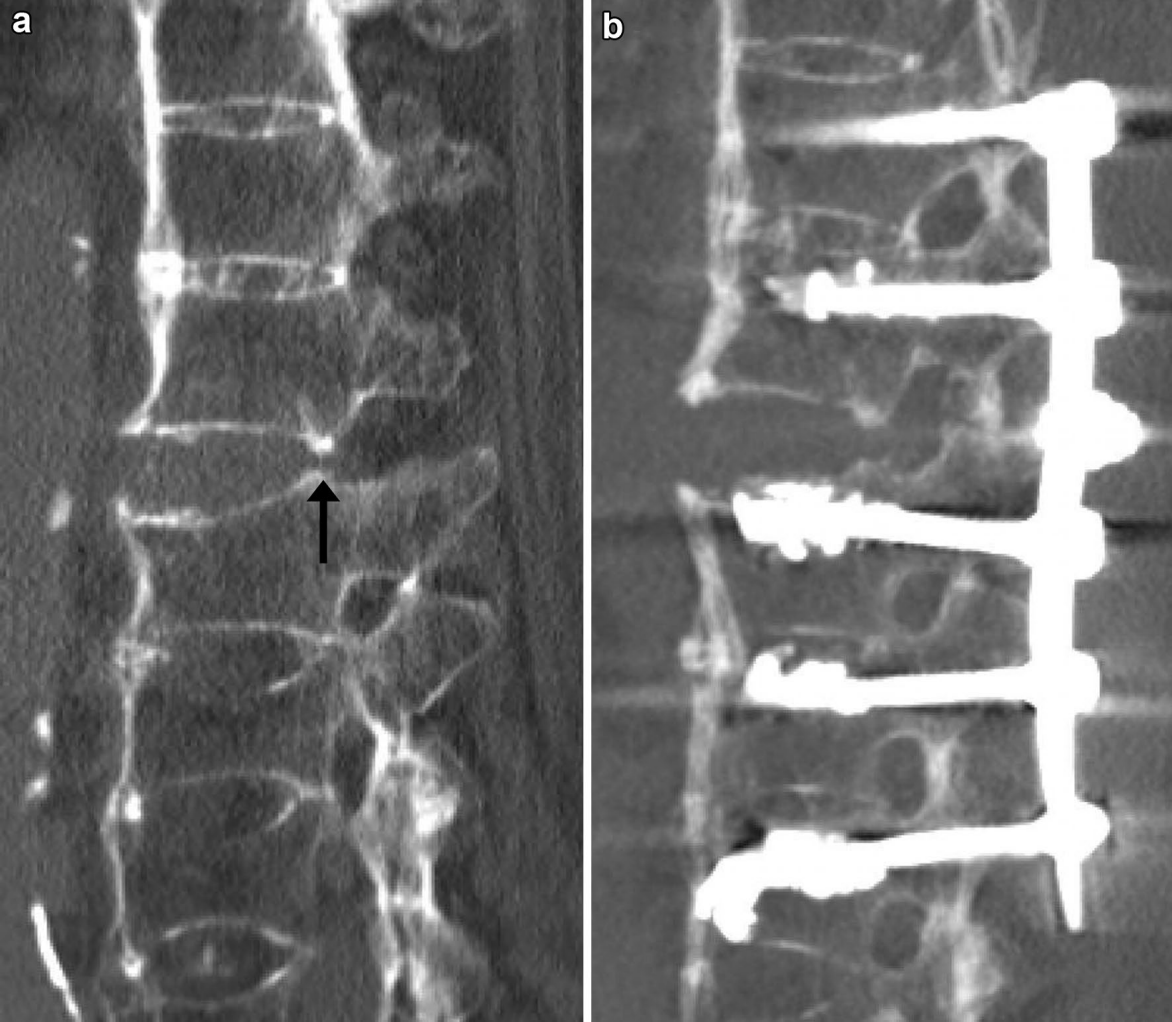
81-year-old male, hyperextension injury of Th12/L1, 13° of lordotic angulation in the pre-operative CT scan (**a**). Postoperative CT scan prior to mobilisation after cement-augmented open rigid posterior instrumentation. No relevant intraoperative reduction of lordotic angulation (**b**)

Besides decreasing the risk of implant loosening, the use of soft rods may be a solution for an additional relevant issue in these patients, and that is fracture reduction and restoration of pre-injury sagittal alignment. While prone positioning facilitates the reduction of kyphotic malalignment in the much more common thoracolumbar compression as well as flexion-distraction injuries, this does not hold true for hyperextension injuries which come along with lordotic malalignment. Intraoperative prone positioning of the patient (which is the only way of positioning to allow for pedicle screw insertion and posterior instrumentation) rather impedes reduction of lordotic angular displacement associated with hyperextension injuries. In our experience, efforts to modify intraoperative prone positioning to facilitate reduction of lordotic angular displacement are usually ineffective and without success. We, for example, tried various strategies of padding below the patient at the level of the apex of lordotic angulation, but this did only marginally help to improve fracture reduction and regularly resulted in substantial intraoperative ventilation problems in these commonly aged and often multimorbid patients due to an increase of intraabdominal pressure. Furthermore, brisk reduction manoeuvres additionally may lead to loosening of the pedicle screws and bears the risk of iatrogenic neurological deficits in these patients [8]. Thus, reduction of hyperextension injuries of the ankylosed spine is extremely challenging; in many cases, no adequate reduction can be achieved by the conventional intraoperative reduction strategies, and despite all reduction efforts, a residual lordotic angular displacement often cannot be avoided. Our concept, therefore, includes flat prone intraoperative positioning of the patient and stabilisation of the fracture in this position without actively performing fracture reduction (Figs. 1b, 5b). Postoperative mobilisation then induces bending of the rods and consecutive fracture reduction (Fig. 2) and thus restores pre-injury sagittal alignment. Our data show that fracture reduction by postoperative mobilisation occurs within the first 3 weeks, when using soft rods (PLR group), and within 6 months with the use of more rigid rods (OR group) (Table 4; Figs. 2, 3). Rapid postoperative reduction with the use of soft rods is highly desirable, as reduction not only promotes osseous healing of the fracture by adding compression to the fracture site (Fig. 4), but also increases the stability of the construct by restoring the buttress function of the anterior column of the spine, therefore, decreasing the risk of pedicle screw loosening and facilitating mobilisation of the patient. This additionally argues for the use of soft rods for posterior instrumentation of hyperextension injuries of the thoracolumbar spine in patients with ankylosing spinal disorders. Percutaneous less rigid instrumentation allowed to restore pre-injury sagittal alignment in all of our patients and osteotomy secondary to trauma was necessitated in none of them.

While our concept of postoperative reduction by mobilisation may be new for the treatment of spinal fractures, it is well known in the treatment of shaft fractures of the upper and lower extremity. For example, dynamic locking of intramedullary nails results in compression of the fracture site during postoperative weight-bearing. This concept requires that the direction of axial compression is provided by the nail and the amount of shortening is limited by the cortical contact between the main fragments. Accordingly, dynamic locking is particularly advisable in simple transverse fractures, while comminuted fractures with no cortical contact between the main fragments require static locking. In hyperextension injuries, the instrumentation is aimed to prevent translation and an increase in lordotic angulation, but, in contrast to compression injuries, does not have to protect the anterior column against compressive forces as the vertebral body is not comminuted but horizontally disrupted. In our concept, osseous contact at the fracture site restores the buttress function of the anterior column of the spine and limits further bending of the rods. The bending should additionally be directed by intact posterior walls, which act as a fulcrum (arrows in Figs. 1a, 5a). The implant itself avoids translational displacement of the fracture during mobilisation to prevent spinal cord encroachment. Accordingly, we do not recommend the use of our concept in fractures with translational displacement or distraction, which disallows the posterior walls to act as a fulcrum, and in fractures with comminution of the anterior column, which is, however, very rare in hyperextension injuries.

Another difference between the two techniques used in this study is the open vs. percutaneous approach. An increased perioperative blood loss during spinal surgery in patients with ankylosing disorders has been described in several studies [8, 21]. It is reasonable to assume that a percutaneous approach may be beneficial in terms of reducing intraoperative blood loss particularly for multisegmental instrumentation of the spine. Due to the retrospective nature of this study, however, we were not able to assess the amount of blood loss in our patients collective. In addition, the risk of impaired wound healing and infection is relatively high in these patients. Backhaus, for example, reports an infection rate of 14% [18]. Atrophic and degenerative trunk muscles as a result of inactivity due to the ankylosed spine may account for this finding. We had a relative risk of impaired wound healing of 21.4% (3/14) after performing an open procedure (OR group), while there was no case of impaired wound healing in the percutaneous group (PLR group). A potential drawback of a percutaneous procedure is a higher rate of pedicle screw misplacement, as there is no clinical control of pedicle screw insertion using landmarks, but radiological control only. The clinical landmarks, however, may be hard to identify even after using an open approach especially in patients suffering from ankylosing spondylitis with ossification of the posterior ligaments and joints. In our study, there was no significant higher rate of pedicle screw misplacement in the PLR group (12.5 vs 9.2%, *p* = 0.78, Table 2). We, therefore, think that percutaneous pedicle screw placement is safe in these patients when performed by surgeons, who are familiar with the procedure.

Our data show that the patients in the PLR group had less non-surgical postoperative complications (Table 3) and a shorter length of hospital stay despite more co-morbidities according to the Charlson Index. We explain these findings by the lower rate of surgical complications and the smaller surgical trauma due to the percutaneous approach in this group. An interesting finding of our study is that clinical outcomes at final follow-up were very good in both groups. The loss in VAS Spine Score, Parker Mobility Score, and Barthel Index (as compared to pre-operative level) was only minimal (Table 5). This is in contrast to studies assessing, for example, the outcome after thoracolumbar burst fractures. Knop reported an average VAS Spine Score loss after combined posterior–anterior stabilisation of 19.7 points [22], while Reinhold even found a loss of 32.2 points after non-operative treatment [23]. In our study, the VAS score loss was 0.9 and 3.2 points, respectively. These findings may be explained by both the lower demand in a predominant geriatric patient population and by the fact that thoracolumbar burst fractures lead to loss of motion segments, while fracture healing restores the pre-traumatic state in patients with ankylosing spinal disorders.

This study has several strengths worth mentioning. First, and in contrast to the previous reports, our study comprises a relatively high number of patients and includes a control group, whereas the currently available literature on hyperextension injuries of the ankylosed thoracolumbar spine is limited to case reports and small case series solely describing patients’ characteristics, mixing up patients with and without neurological deficits and often without specifying surgical stabilisation. Moreover, these studies are lacking a control group as well as a comparison of different treatment strategies. Second, our study not only reports radiological but also clinical outcomes assessed by a set of validated outcome scores to quantify pain and function, mobility, social independency, and quality of life after a respectable mean follow-up period of 29.2 months, the longest reported so far. Third, we proposed a new treatment approach for these rare but clinically challenging injuries and, for the first time, comprehensively outlined and discussed important aspects to be considered when choosing the optimal treatment strategy for these patients.

Some limitations of our study, however, have to be noted. First, this is a retrospective study with all limitations associated with this study design. For example, we were not able to retrospectively assess the amount of intraoperative blood loss. Second, a higher number of patients would have increased the power of the statistical analysis and may have revealed more significant differences between the two groups; however, given the rarity of this injury pattern, small sample size is a limitation inherent to studies addressing thoracolumbar fractures in patients with ankylosing spinal disorders [24]. Small sample size in this study is, furthermore, a result of exclusion of patients with neurological deficits and additional fractures which was inevitable to be able to compare clinical outcome between groups. In addition, there were more patients in the OR group than in the PLR group. The authors, therefore, are aware that the data of this study cannot form a sound basis to guide treatment approaches, but, nevertheless, represent a “proof of principle” of a new concept to address this type of injury, considering the specific biomechanical characteristics of the ankylosed thoracolumbar spine. Third, the choice of instrumentation did not follow institutional guidelines, but was made by the involved surgeon. Fourth, functional scores (i.e. VAS Spine Score, Parker Mobility Score and Barthel Index) for the time prior to the injury were assessed at final follow-up. Finally, patients with two different ankylosing spinal disorders (AS and DISH) were included in the study. Besides several differences in aetiology and radiographic appearance, however, these two ankylosing spinal disorders share surgically relevant clinical and biomechanical features, which legitimates the merging of both diseases into one study group for a surgery-related study [6].

## Conclusions

The results of this patient series indicate that our new treatment concept for the surgical management of hyperextension injuries of the thoracolumbar spine in patients with ankylosing spinal disorders is feasible; seems to facilitate early and adequate fracture reduction; might have the potential to reduce the rate of complications; and results in comparable outcome as the conventional open rigid instrumentation. The proposed treatment concept involves flat prone positioning of the patients; fracture fixation without actively performing reduction of the lordotic angular displacement; and multisegmental percutaneous posterior instrumentation using less rigid rods to promote postoperative fracture reduction and restoration of pre-injury sagittal alignment by postoperative mobilisation. However, in patients with additional translational displacement or distraction of the fracture as well as in the very rare cases of hyperextension injuries coming along with vertebral body comminution, we still consider an open rigid posterior instrumentation to be the preferable treatment strategy.
